# High glucose inhibits autophagy and promotes the proliferation and metastasis of colorectal cancer through the PI3K/AKT/mTOR pathway

**DOI:** 10.1002/cam4.7382

**Published:** 2024-06-13

**Authors:** Feng Li, Xing Wan, Zhigui Li, Liming Zhou

**Affiliations:** ^1^ Department of Pharmacology West China School of Basic Science and Forensic Medicine, Sichuan University Chengdu China; ^2^ Department of General Surgery West China Hospital, Sichuan University Chengdu China

**Keywords:** autophagy, colorectal cancer, high‐glucose, PI3K

## Abstract

**Background:**

Colorectal cancer (CRC) ranks among the most prevalent malignancies worldwide, characterized by its complex etiology and slow research progress. Diabetes, as an independent risk factor for CRC, has been widely certified. Consequently, this study centers on elucidating the intricacies of CRC cells initiation and progression within a high‐glucose environment.

**Methods:**

A battery of assays was employed to assess the proliferation and metastasis of CRC cells cultured under varying glucose concentrations. Optimal glucose levels conducive to cells' proliferation and migration were identified. Western blot analyses were conducted to evaluate alterations in apoptosis, autophagy, and EMT‐related proteins in CRC cells under high‐glucose conditions. The expression of PI3K/AKT/mTOR pathway‐associated proteins was assessed using western blot. The effect of high glucose on xenograft growth was investigated in vivo by MC38 cells, and changes in inflammatory factors (IL‐4, IL‐13, TNF‐α, IL‐5, and IL‐12) were measured via serum ELISA.

**Results:**

Our experiments demonstrated that elevated glucose concentrations promoted both the proliferation and migration of CRC cells; the most favorable glucose dose is 20 mM. Western blot analyses revealed a decrease in apoptotic proteins, such as Bim, Bax, and caspase‐3 with increasing glucose levels. Concurrently, the expression of EMT‐related proteins, including N‐cadherin, vimentin, ZEB1, and MMP9, increased. High‐glucose cultured cells exhibited elevated levels of PI3K/AKT/mTOR pathway proteins. In the xenograft model, tumor cells stimulated by high glucose exhibited accelerated growth, larger tumor volumes, and heightened KI67 expression of immunohistochemistry. ELISA experiments revealed higher expression of IL‐4 and IL‐13 and lower expression of TNF‐α and IL‐5 in the serum of high‐glucose‐stimulated mice.

**Conclusion:**

The most favorable dose and time for tumor cells proliferation and migration is 20 mM, 48 h. High glucose fosters CRC cell proliferation and migration while suppressing autophagy through the activation of the PI3K/AKT/mTOR pathway.

## 1 INTRODUCTION

Colorectal cancer (CRC) is one of the malignant tumors with high incidence and mortality in the world.^1^ The etiology of colorectal cancer is multifactorial, including factors such as dysbiosis of the gut microbiota, genetic mutations, underlying medical conditions, unhealthy lifestyles, among others^.^ ^2^, ^3^, ^4^, ^5^, ^6^, ^7^ Among these underlying medical conditions, a close relationship has been established between diabetes and colorectal cancer. Several studies have indicated that diabetes has emerged as an independent risk factor for CRC, contributing to adverse prognostic outcomes in terms of overall mortality, cancer‐specific mortality, and disease‐free survival rates associated with CRC.^8^, ^9^, ^10^ Research has shown that CRC patients with diabetes experience a reduced lifespan of up to 5 years and an overall survival rate decrease of 18% compared to those without diabetes.^11^ Based on currently available data, diabetes has indeed become a prevalent chronic disease. In China, the prevalence of diabetes among adults was 12.4% in 2018, and it is projected to increase to 18.5% by 2030.^12^ Therefore, investigating the mechanistic link between diabetes and CRC in this context holds significant clinical relevance.

Autophagy is a universally occurring cellular degradation process in response to energy stress. When exposed to external stimuli, cells transport damaged organelles or large molecular substances to lysosomes for degradation and digestion.^13^ Autophagy is generally divided into three categories, namely macroautophagy, microautophagy, and chaperone‐mediated autophagy (CMA).^14^ Following initiation of autophagy within the cells, under the regulation of autophagy‐related genes, cells envelop the cytoplasm or organelles to be degraded with a single or double membrane, forming a double‐membrane precursor structure known as an autophagosome. Subsequently, the autophagosome fuses with a lysosome, resulting in the formation of an autolysosome. Within the lysosome, hydrolytic enzymes degrade the contents enclosed by the autophagosome.^15^, ^16^ This process enables cells to renew their energy and metabolism, contributing to cellular homeostasis.

PI3K is a lipid kinase in cells that regulates the proliferation and differentiation of cancer cells.^17^, ^18^ The PI3K/AKT/mTOR pathway is currently a hot topic of research, and PI3K/AKT/mTOR signaling pathway plays a crucial role in the processes of tumor proliferation, metastasis, survival, and angiogenesis.^19^ At the same time, this pathway is closely related to autophagy, and many drugs achieve the goal of inhibiting tumor proliferation by inhibiting this pathway.^20^, ^21^, ^22^, ^23^, ^24^ mTOR is the central checkpoint that negatively regulates autophagy, and anti‐cancer drugs weaken the PI3K/AKT/mTOR pathway to stimulate autophagy. High‐glucose levels have been shown to promote the proliferation of various types of tumors, such as breast cancer,^25^ CRC,^26^ lung cancer,^27^ and liver cancer.^28^ Tumor cells exposed to high glucose exhibit intracellular signaling alterations that lead to a more aggressive phenotype.^28^, ^29^, ^30^ Although high glucose can provide tumor cells with the additional nutrients they require for growth, it is not that higher glucose is better. Therefore, determining the highest glucose concentration that tumor cells can adapt to is an intriguing area of research. In this study, we investigated the impact of different glucose concentrations on the proliferation and migration abilities of CRC cells. We identified the optimal concentration that favors CRC cell growth and examined changes in relevant molecular markers under high‐glucose conditions. Furthermore, we confirmed that high glucose can promote tumor cell proliferation and migration through the PI3K/AKT/mTOR pathway.

## 2 MATERIALS AND METHODS

### 2.1 Cell culture

HCT116, MC38, and DLD1 cells were purchased from American Type Culture Collection (ATCC, Manassas, VA, USA). Glucose (Solarbio, China) was diluted to 1 M with DMEM, chloroquine (CQ, MCE, USA), and PI3K inhibitor LY294002 (MCE, USA).

### 2.2 CCK8 assay

HCT116, DLD1, and MC38 cells were inoculated in 96‐well plates, adjusted to 1000/well, with varying glucose concentrations in the respective culture medium. Following seeding, they were subjected to continuous cultivation for 24 and 48 h, respectively. Subsequently, the culture medium was aspirated and replaced with a solution of DMEM: CCK8 (9:1). Subsequent absorbance measurements were performed using an enzyme‐linked immunosorbent assay reader.

### 2.3 Colony formation

HCT116, DLD1, and MC38 cells were inoculated in 6‐well plates that seeded at a density of 1000/well, and the medium with different glucose concentration was changed every 3 days. The cells were cultured continuously for 14 days. After the 14‐day incubation, added 1 mL fixing solution to each well, let stand at room temperature for 30 min. Then, the cells with 5% crystal violet stain 30 min. After staining, the wells were lightly washed with PBS and photographed.

### 2.4 Cell migration and invasion assays

Trypsin was used to digest HCT116, DLD1, and MC38 cells and count the cells. Medium containing 10% serum and different glucose concentrations were added into the lower chamber of the 24‐well plate. A matrix glue was pre‐coated in the upper chamber 3 h in advance (matrix glue: DMEM = 1:8, this step was left out in the migration experiment), and 4 × 10^4^ cells were added into the upper chamber (there was no serum but glucose in the upper chamber) and the cells were cultured with 5% CO_2_ at 37°C. After 24/48 h, the chamber was removed, fixed with 5% paraformaldehyde for 30 min, and then stained with 5% crystal violet for 30 min. The excess dye liquid in the chamber was cleaned with PBS, and the water in the chamber was carefully wiped with cotton swabs. They were allowed to dry and photographed with a microscope (Leica, Germany) and counted using Image J.

### 2.5 Wound healing assay

Three horizontal lines were drawn on the back of the 6‐well plate, and 3 × 10^5^/well was inoculated into the each well and cultured overnight in the cell incubator. The next day, a 200 μL pipette tip was used to draw lines perpendicular to the back of the 6‐well plate, and the floating cells were gently washed with PBS. At the same time, an appropriate amount of the storage solution was taken to prepare medium with different glucose concentrations (without FBS) and each well's corresponding concentration of medium was added. At 0, 24, and 48 h, photographs were taken by microscope (Leica, Germany) and analyzed by Image J.

### 2.6 Western blot

The treated cells were washed with PBS for 3 times, lysed in RIPA buffer (contained PMSF) for 30 min during the cells were thoroughly mixed by vortex oscillation, and centrifuged for 13,000 ***g*** and 10 min after the lysate being completed. Keep the supernatant stay and add proper loading buffer at 100°C for 10 min. Appropriate amount of RIPA lysate (contained PMSF) was added to the tumor tissue for cleavage using a tissue homogenizer (60 Hz, 3 min), and the lysate was left on ice for 30 min, and then, the procedure was the same as that of cell protein extraction. Protein concentration was determined by BCA, and the supernatants were loaded onto SDS‐PAGE gels and then transferred onto NC membranes (PALL, USA). After the membrane was transferred, 5% skim milk was used for sealing for 2 h, and then, primary antibody was added and kept at 4°C overnight (dilution ratio of antibody was showed in Table S1). TBST was cleaned three times for 10 min each time, and then, secondary antibodies were added and incubated in a shaker for 90 min. After incubation, TBST was cleaned three times for 10 min each time. Immunoreactive proteins were performed using a chemiluminescence apparatus (CLINX 6200Touch). Image J was used for grayscale analysis.

### 2.7 Ad‐mCherry‐GFP‐LC3B transfect

Slides were added to 24‐well plates; HCT116 cells were seeded on the cell slides for 24 h and then washed three times with PBS, and AdPles‐mCherry‐GFP‐LC3B adenovirus was added to infect the cells at an MOI of 40. After an additional 24 h of incubation, the cells were washed with PBS and cultured with 0 and 20 mM glucose‐containing medium for 48 h. Similarly, 4% paraformaldehyde was fixed for 30 min, and then, DAPI was used to dye the nucleus for 5 min, and the expressions of DAPI, mCherry, and GFP were observed by confocal laser scanning microscope (Zeiss, LSM710, Germany).

### 2.8 Animal experiments

Eight C57 mice (4–6 weeks old) were fed acclimatization at animal center, where they were free to drink water and eat food, and they were randomly divided into two groups with four mice in each group. MC38 cells were adjusted to a concentration of 1 × 10^6^ cells in 100 μL and subcutaneously injected into the thigh root of the mice. The mice's general condition was observed daily, and every 48 h, they were intraperitoneally injected with 200 μL of a 20 mM glucose solution (prepared in physiological saline), while the control group received an equivalent volume of physiological saline. Tumor size was measured and recorded every other day, and a tumor growth curve was plotted. Mice were euthanized, and tumors were excised, photographed, fixed in 5% paraformaldehyde, and subsequently processed into paraffin blocks. The tumor volume was calculated according to the following equation: *V* = (length × width^2^)/2. All animal experiments were approved by the Institutional Animal Care and Use Committee of Sichuan University.

### 2.9 ELISA assay

In total, 200 μL of orbital sinus blood was taken before sacrificed. After standing for 4 h and centrifugation at 1000 ***g*** for 10 min, about 100 μL of serum was obtained. The sera were harvested and pooled for IL‐4, IL‐13, TNF‐α, IL‐5, and IL‐12 analysis with an ELISA kit (MultiSciences, China), which was used according to the manufacturer's instructions.

### 2.10 Statistical analysis

All in vitro experiments were repeated three times. The data were analyzed by one‐way anova or Student's *t* test, and software usage includes SPSS 25.0 (SPSS, Chicago, IL, USA) GraphPad Prism 8.0 (GraphPad software, La Jolla, CA, USA), and Image J (NIH, USA).

## 3 RESULTS

### 3.1 High glucose promotes tumor growth

**FIGURE 1:**
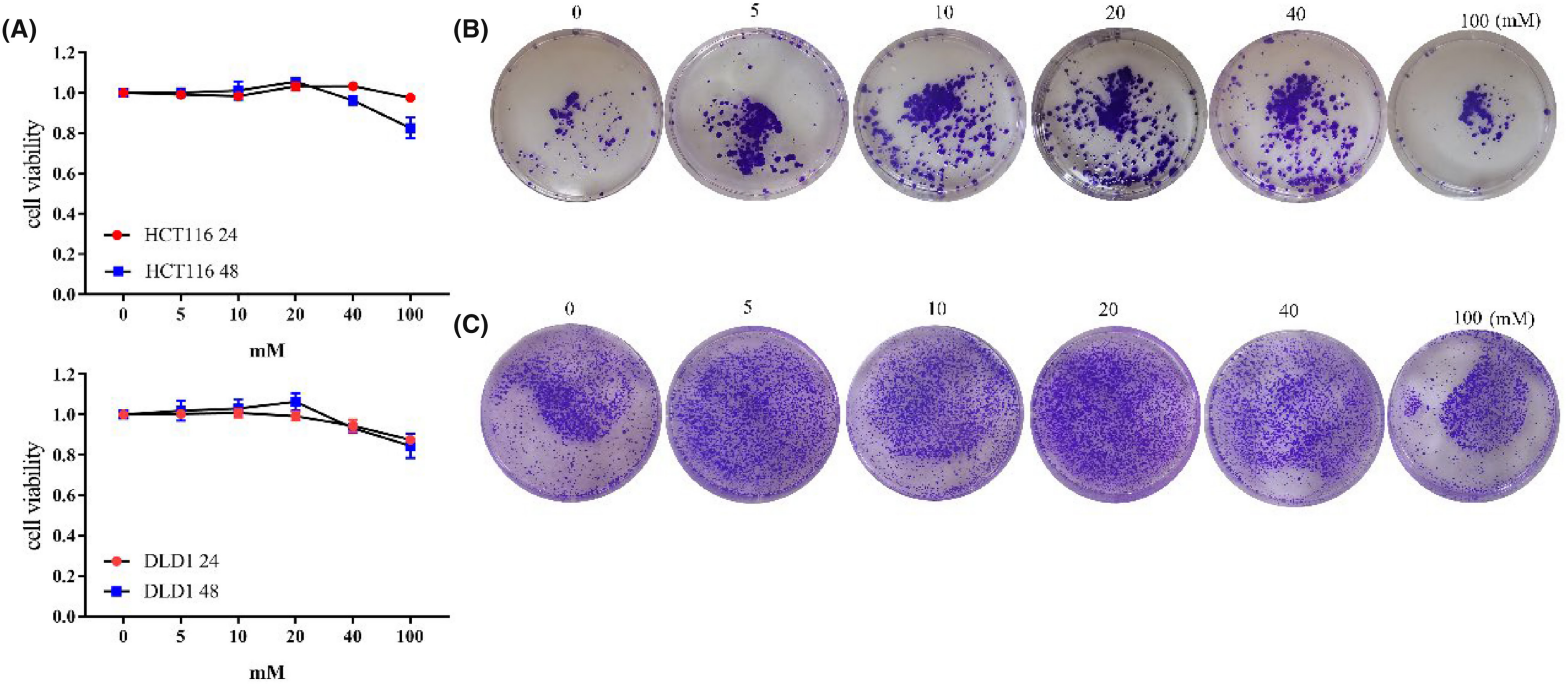
High glucose promotes tumor cell proliferation. (A) The activity of tumor cells stimulated with different glucose concentrations by CCK‐8. Top: HCT116, Bottom: DLD1. (B) Colony formation assay of HCT116 which cultured with different glucose concentrations. (C) Colony formation assay of DLD1 which cultured with different glucose concentrations.

Firstly, we investigated the impact of different glucose concentrations on tumor cell proliferation. Within the range of 0‐20 mM, as glucose concentration increased, cell proliferation capacity also increased. This trend was consistent at both 24 and 48 h. Both HCT116 and DLD1 cells exhibited the highest proliferation capacity at 20 mM 48 h. HCTT16 showed a slight decrease in proliferation capacity at 40 and 100 mM after 24 h, with a more pronounced decrease observed at 48 h. DLD1 also exhibited decreased proliferation capacity at 40 and 100 mM after both 24 and 48 h (Figure 1A, Figure S1A). Colony formation assay provided a visual representation of changes in proliferation. With increasing glucose concentration, there was an increase in the number of cells colonies formed. The highest number of colonies was observed at 20 mM (Figure 1B,C, Figure S1B). Therefore, the cell proliferation capacity was the strongest at 20 mM glucose concentration.

### 3.2 High‐glucose levels enhance tumor metastatic ability

**FIGURE 2:**
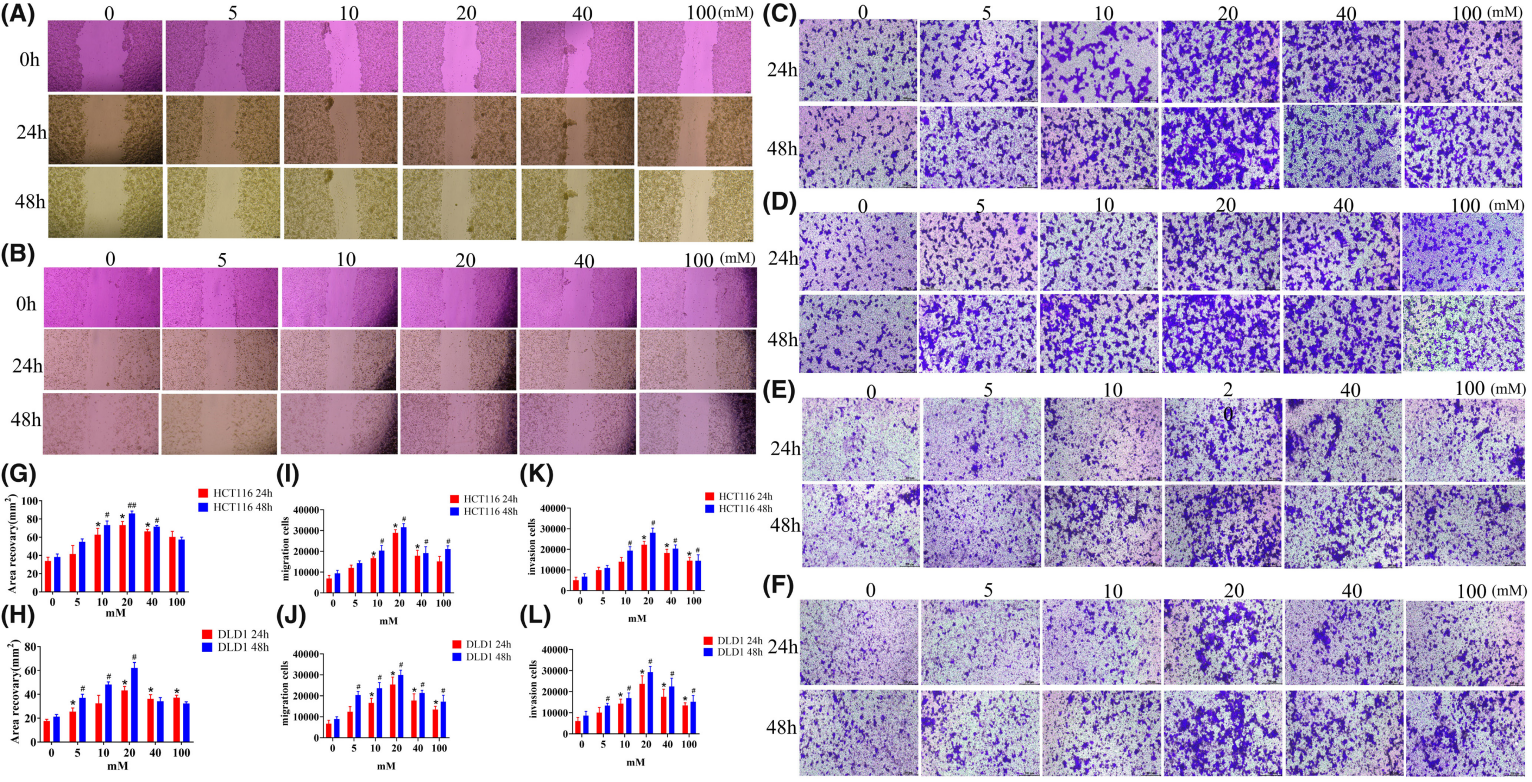
High glucose promotes tumor cell migration. (A) Wound healing assay in HCT116 cells under different glucose concentrations. (B) Wound healing assay in DLD1 cells under different glucose concentrations. (C) Transwell migration experiment in HCT116 cells under different glucose concentrations. (D) Transwell migration experiment in DLD1 cells under different glucose concentrations. (E) Invasion assay of HCT116 cells under different glucose concentrations. (F) Invasion assay of DLD1 cells under different glucose concentrations. (G, H) Statistical graphs of wound healing assay for HCT116 and DLD1 cells. (I, J) Statistical graphs of transwell migration assay for HCT116 and DLD1 cells. (K, L) Statistical graphs of invasion assay for HCT116 and DLD1 cells. The values are presented as the mean ± SD of three independent experiments, *p < 0.05, vs. 0 mM 24 h, #*p* < 0.05, ##*p* < 0.01 vs. 0 mM 48 h.

Similar to the proliferation experiments, we conducted related experiments using different glucose concentrations. At 24 h, as the glucose concentration increased, cell migration ability increased between 0 and 20 mM, although migration ability increased at 40 and 100 mM, it slightly decreased compared to 20 mM. The trend was consistent at 48 h. The changes observed in HCT116, DLD1, and MC38 cells were consistent (Figure 2A,B,G,H, Figure S2A).

In migration and invasion experiments, as the glucose concentration increased, cell migration and invasion abilities gradually increased between 0 and 20 mM at 24 h, while at 40 and 100 mM, migration ability decreased. At 48 h, the number of migrating cells in all six groups was higher than at 24 h, but the maximum was observed at 20 mM (Figure 2C–F,I–K, Figure S2C,D). Therefore, at 48 h, cells exhibited the strongest migration and proliferation abilities at 20 mM.

### 3.3 High glucose inhibits apoptosis‐related proteins and promotes the expression of EMT‐related proteins

In conjunction with the previous observations on the effects of different glucose concentrations on tumor cell proliferation and migration, we chose a final cultivation concentration and time at 20 mM of 48 h. To observe the trends in protein expression, we also selected an intermediate concentration of 5 mM for the detection of relevant proteins.

**FIGURE 3:**
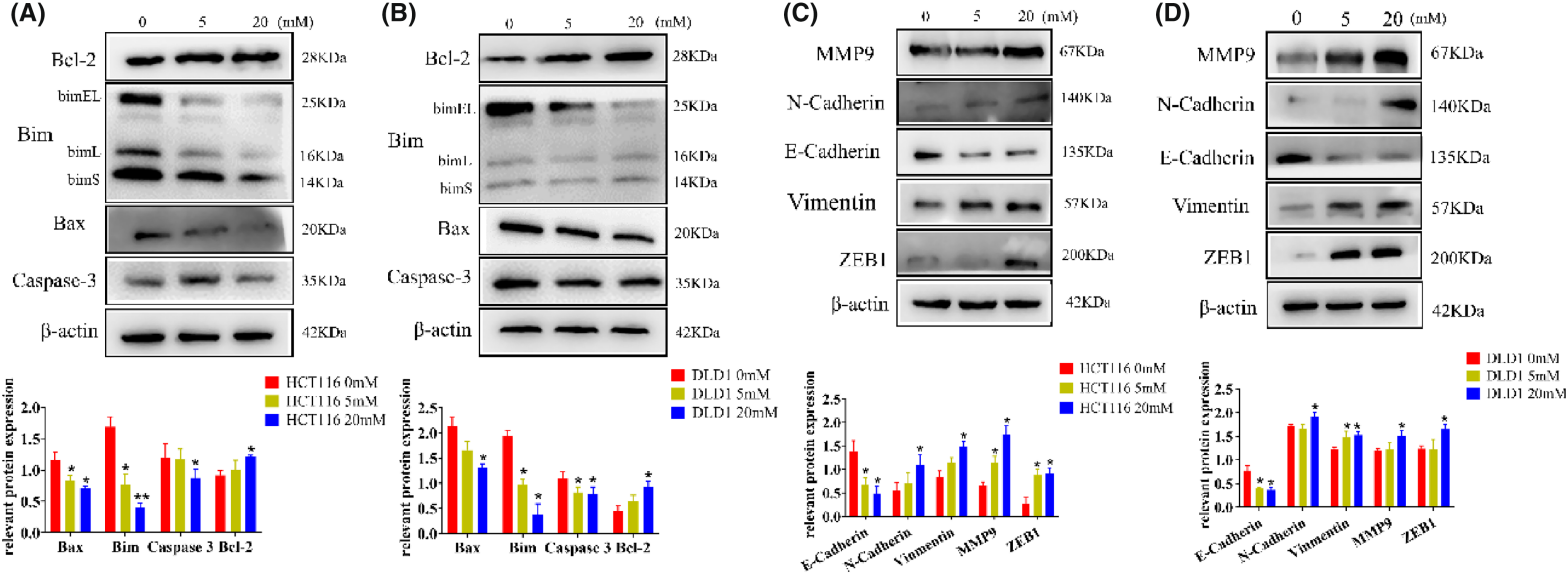
High glucose suppresses apoptosis‐related protein expression and promotes EMT‐related protein expression. (A) The expression of apoptosis‐related factors inhibited by high glucose was detected by western blot in HCT116 cells. (B) The expression of apoptosis‐related factors inhibited by high glucose was detected by western blot in DLD1 cells. (C) The expression of EMT‐related protein promoted by high glucose was detected by western blot in HCT116 cells. (D) The expression of EMT‐related protein promoted by high glucose was detected by western blot in DLD1 cells. The values are presented as the mean ± SD of three independent experiments, *p < 0.05, **p < 0.01 vs. 0 mM.

From the results, it is evident that the expression of apoptosis‐related proteins such as Bax, Bim, and caspase 3 decreased, while the anti‐apoptotic protein Bcl‐2 increased, with a particularly noticeable decrease in Bim expression (Figure 3A,B, Figure S3A). High‐glucose levels influenced the migration of tumor cells, prompting the examination of (epithelial‐mesenchymal transition) EMT‐related proteins. It is observed from the results that the protein expression of MMP9, N‐cadherin, vimentin, and ZEB1 increased, while E‐cadherin protein decreased (Figure 3C,D, Figure S3B). The changes in the expression of EMT‐related proteins were prominently evident.

### 3.4 High glucose regulates apoptosis by inhibiting autophagy

**FIGURE 4:**
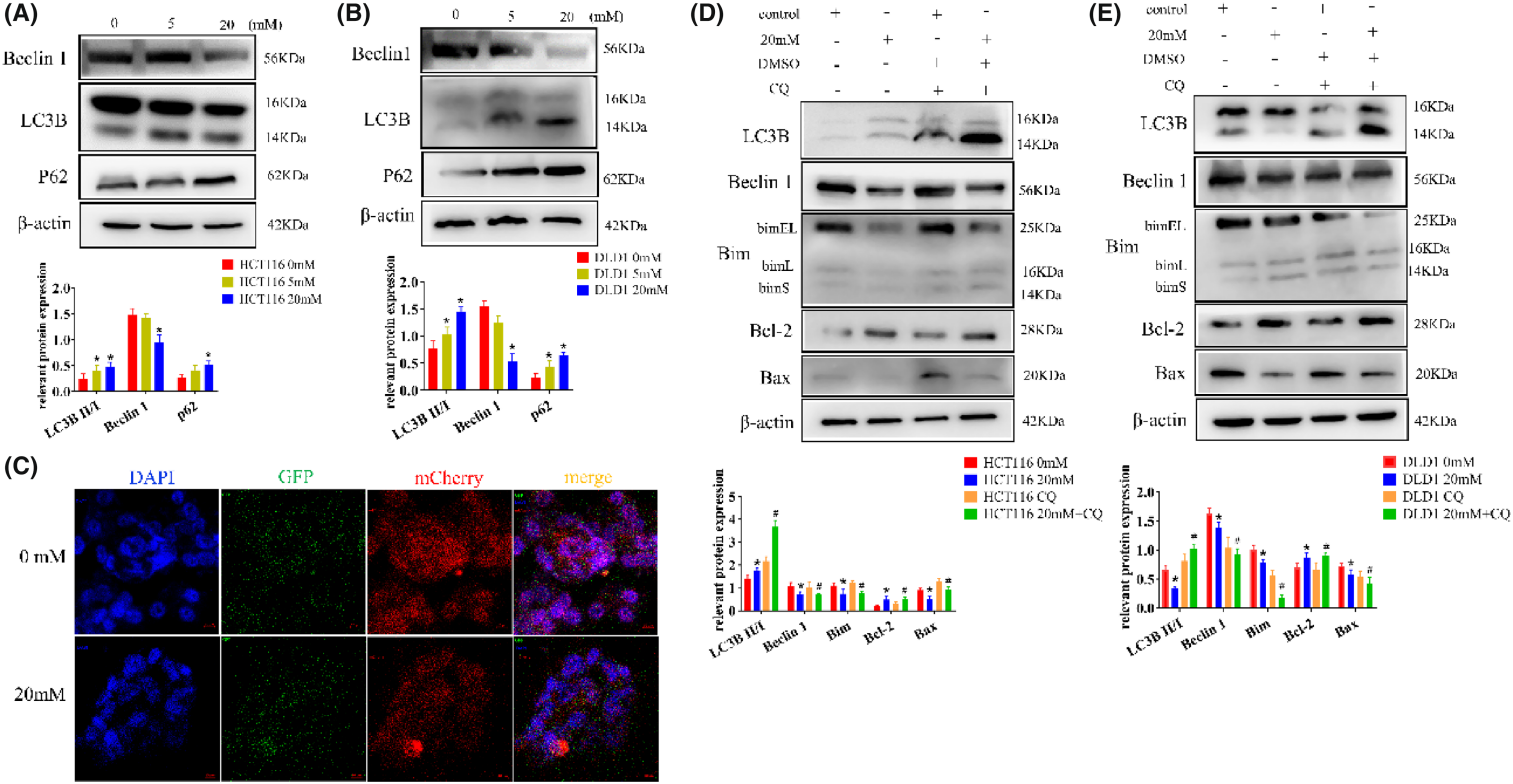
High glucose regulates apoptosis by inhibiting autophagy. (A) The expression of autophagy‐related proteins inhibited by high glucose was detected by western blot in HCT116 cells. (B) The expression of autophagy‐related proteins inhibited by high glucose was detected by western blot in DLD1 cells. (C) Transfection of HCT116 cells with Ad‐mCherry‐GFP‐LC3B. (D) The expression of autophagy and apoptosis‐related proteins was detected by western blot in HCT116 cells with CQ. (E) The expression of autophagy and apoptosis‐related proteins was detected by western blot in DLD1 cells with CQ. The values are presented as the mean ± SD of three independent experiments, *p < 0.05, vs. 0 mM, #*p* < 0.05, vs. 0 mM + CQ.

Western blot analysis revealed that the expression of beclin1 decreased, the ratio of LC3B II/I increased in the high‐glucose group, and P62 expression also increased compared with the control (Figure 4A,B, Figure S4A). Since we have observed these trends and combined them with the results of experiments involving apoptosis‐related factors and EMT‐related proteins, we identified 20 mM as the critical concentration at which high glucose alters the behavior of tumor cells. Consequently, we chose 20 mM for our subsequent experiments.

To observe autophagic flux, we employed Ad‐mCherry‐GFP‐LC3B for lentiviral transduction. Notably, the high‐glucose group exhibited stronger green fluorescence, which appeared yellow when overlaid with red fluorescence. This indicates a clear inhibitory effect of high glucose on autophagic flow in HCT116 cells (Figure 4C).

Next, in the study of the regulatory relationship between autophagy and CRC cell apoptosis, the cells were treated with the autophagy inhibitor CQ. Firstly, as seen in the figures, within the high‐glucose group, there was an increase in the LC3B II/I ratio. After the addition of CQ, this ratio significantly increased. Simultaneously, the expressions of Beclin1, Bim, and Bax decreased compared to the high‐glucose group alone, with Bcl‐2 expression registering a higher level than in the high‐glucose group alone (Figure 4D,E).

### 3.5 High glucose inhibits CRC cell apoptosis via the PI3K/AKT/mTOR pathway

**FIGURE 5:**
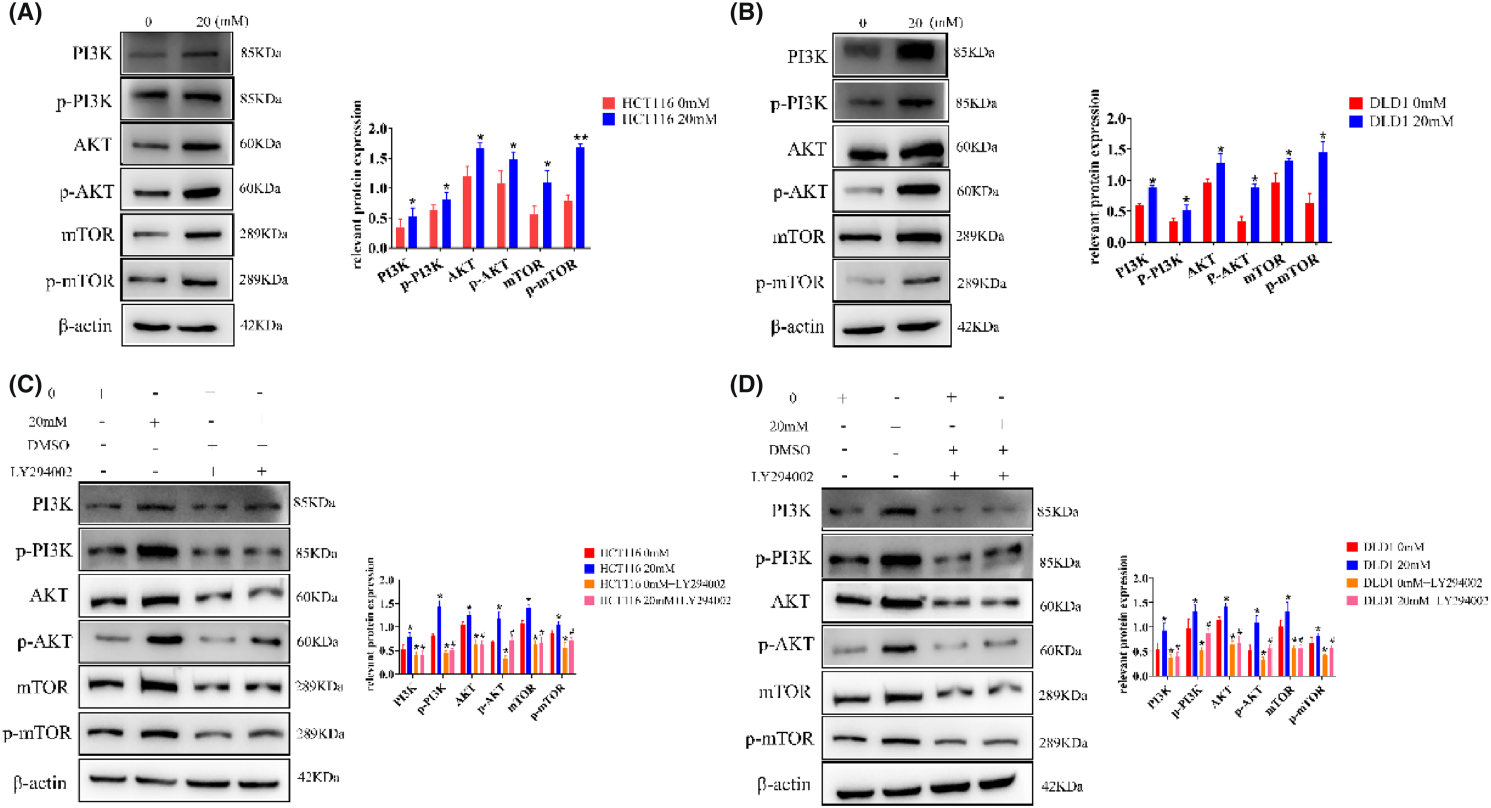
High glucose enhances the expression of PI3K/AKT/mTOR pathway proteins. (A) The PI3K/AKT/mTOR pathway protein was activated by high glucose in HCT116 cells by western blot. (B) The PI3K/AKT/mTOR pathway protein was activated by high glucose in DLD1 cells by western blot. (C) The inhibitor (LY294002) restored activation of the PI3K/AKT/mTOR pathway in HCT116 cells by western blot. (D) The inhibitor (LY294002) restored activation of the PI3K/AKT/mTOR pathway in DLD1 cells by western blot; the values are presented as the mean ± SD of three independent experiments, *p < 0.05, vs. 0 mM; #*p* < 0.05, vs. 20 mM.

Considering a prominent influence of the PI3K/AKT pathway to autophagy and EMT in tumor diseases, we detected the expression of all relevant proteins, the expression of PI3K, p‐PI3K, AKT, p‐AKT, mTOR, and p‐mTOR increased (Figure 5A,B, Figure S4B), showing significant differences compared with control group. However, after the addition of PI3K inhibitors (LY294002), the expression of PI3K, p‐PI3K, AKT, p‐AKT, mTOR, and p‐mTOR was all significantly decreased (Figure 5C,D, Figure S4C).

### 3.6 In vivo validation of high‐glucose promotion of tumor growth

**FIGURE 6:**
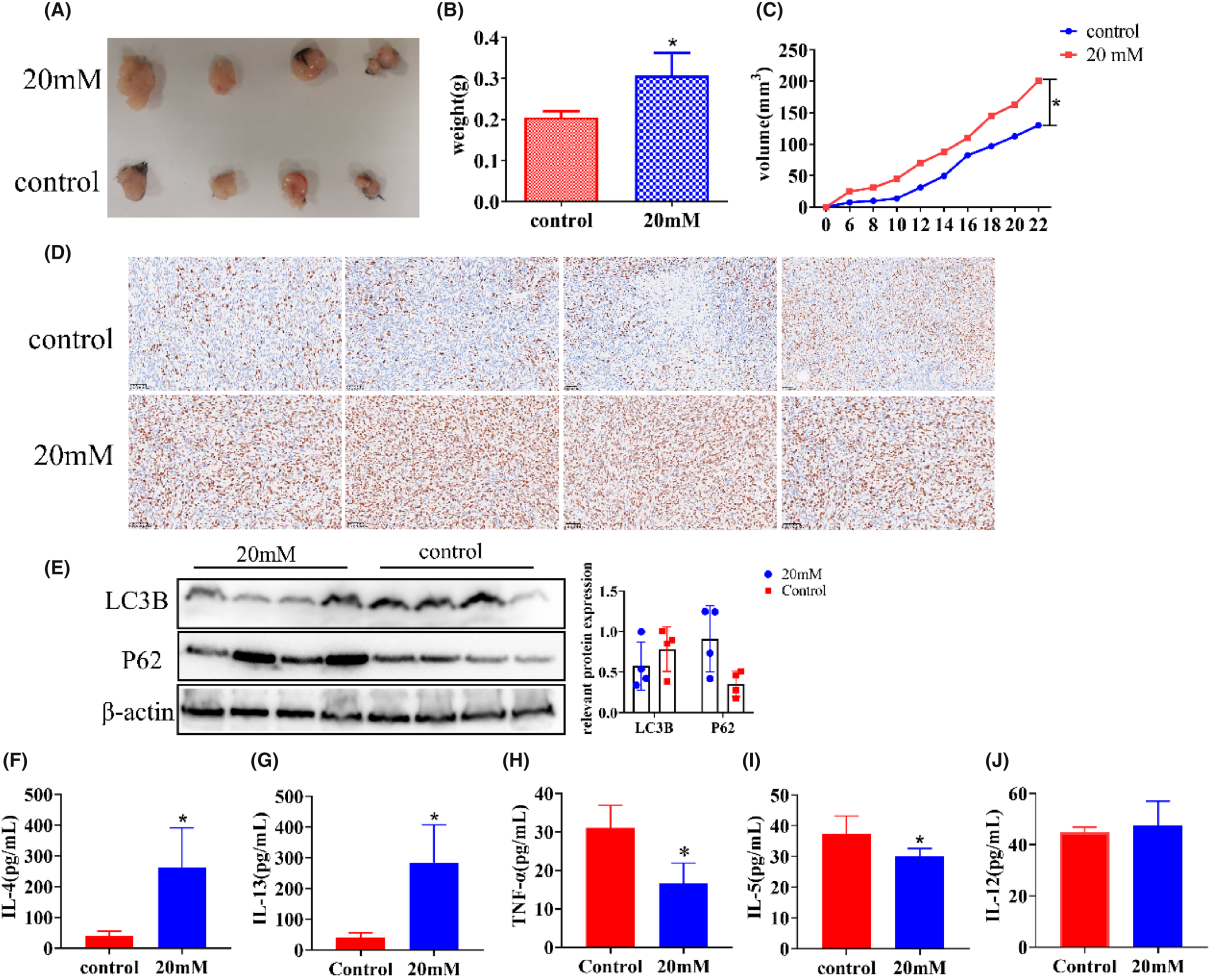
Xenograft growth promoted by high glucose. (A) Effects of high glucose on the tumorigenic ability of MC38 cells. (B) Tumor weight (*P* < 0.05). (C) Tumor growth curve (*P* < 0.05). (D) Higher expression of KI67 protein detected by IHC in tumor tissue of high‐glucose group (400×). (E) The expression of autophagy‐related proteins inhibited by high glucose was detected by western blot in tumor tissues. (F–J) relevant inflammatory factors detected by ELISA (IL‐4, IL‐13, TNF‐α, IL‐5, IL‐12), *p < 0.05, vs. the control group.

**FIGURE 7:**
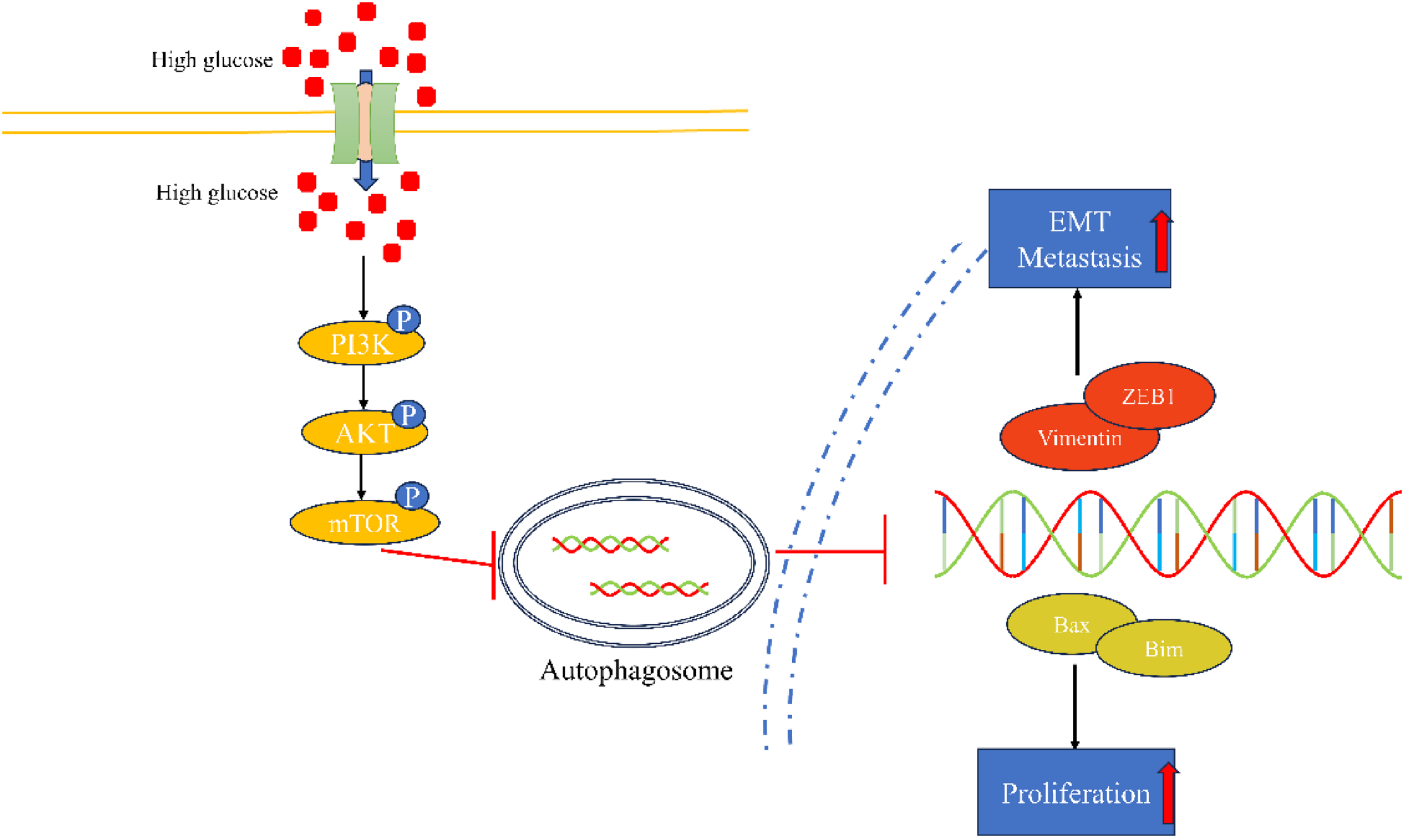
Schematic diagram showing the mechanism by which high glucose promotes CRC progression.

We constructed a xenograft model using C57 mice to observe the effect of high glucose on tumor growth in vivo. It can be seen from the figure that the volume and weight of xenograft in the high‐glucose group were significantly larger (Figure 6A,B), and their growth rate was faster compared to the control group (Figure 6C). In order to visually judge the proliferation degree of the xenograft from the molecular point of view, KI67 staining was performed on the xenograft by immunohistochemistry. The results showed that the expression of KI67 in the high‐glucose group was significantly stronger than that in the control group (Figure 6D). The autophagy level of tumor tissues was detected by western blot; the expression of P62 and LC3B in the high‐glucose group was higher than that in the control group (Figure 6E). Changes in relevant factors in the serum were measured using ELISA. IL‐4 and IL‐13 levels in the experimental groups were markedly higher than those in the control group, with values of 40.26 ± 14.96 vs. 263.2 ± 128.3 and 40.67 ± 15.22 vs. 284.7 ± 122.7, respectively. On the other hand, IL‐5 and TNF‐α levels in the experimental groups were significantly lower than those in the control group, with values of 37.39 ± 5.79 vs. 30.13 ± 2.472 and 31.10 ± 5.834 vs. 16.63 ± 5.258, respectively. However, there was no significant difference in IL‐12 levels between the two groups, with values of 37.39 ± 5.79 vs. 30.13 ± 2.37 (Figure 6F–J). In summary, high glucose inhibits tumor cell autophagy by promoting the PI3K/AKT/mTOR pathway, thus promoting proliferation and metastasis (Figure 7).

## 4 DISCUSSION

CRC has consistently ranked high in terms of incidence and mortality rates, and there are numerous factors contributing to its development, with complex molecular mechanisms involved. Research has already established that diabetes is an independent risk factor for CRC.^31^ Studies have indicated that patients with both CRC and diabetes have a worse prognosis and lower survival rates compared to those with CRC alone.^32^, ^33^, ^34^, ^35^, ^36^, ^37^ Therefore, investigating the proliferation and migration of CRC cells cultured in high‐glucose conditions is of significant importance.

While it is widely accepted that high glucose promotes CRC cell proliferation and inhibits apoptosis, this study has explored the impact of different glucose concentrations on tumor cells. From the experimental results, it can be observed that the promoting effect of high glucose on tumor cells is limited. We believe there are two main reasons for this limitation. Firstly, it is likely that tumor cells experience metabolic stress and altered osmotic pressure in an excessively high‐glucose environment, which may actually become toxic to the cells. Consequently, cell growth is inhibited at a concentration of 100 mM. Secondly, it is possible that the enzymatic reaction rates within the tumor cells have already reached their maximum capacity. Even if more glucose is added, it may not be efficiently converted into energy through metabolism. Therefore, the promotion of tumor growth by high glucose is limited.

Previous studies have shown that high glucose can trigger certain important signaling pathways in CRC.^38^ However, current research on the relationship between the two mainly focuses on pathways related to glucose metabolism. For example, cancer cells show increased dependence on the aerobic glycolysis process with high‐glucose conditions, which also activating other signaling pathways at the same time. As an energy source, glucose not only brings energy for cells to survive but can also participate in the process of energy metabolism, especially the regulation of genes involved in the process of glycolysis, such as GLUT1 and HKII. Glucose can regulate the expression difference of these genes in normal cells and cancer cells that will affect tumor progression.^39^, ^40^ At the same time, some studies have made a preliminary connection between T2DM and CRC. Insulin‐like growth factor receptor (IGFR) can be activated by high‐glucose medium in CRC cells and acts as a messenger to Src and Erk, which promotes the proliferation of CRC cells.^41^ However, there are few studies on the impact of autophagy on CRC cells in a high‐glucose environment. Therefore, the focus of this study is to explore how high glucose affects CRC apoptosis through autophagy.

Autophagy is a cellular process that maintains cellular homeostasis by regularly renewing dysfunctional proteins and organelles at its basal level. It is upregulated in response to cellular stress and nutrient deficiency. Autophagy serves various important roles, including nutrient recycling, preventing the accumulation of misfolded proteins, protecting against damage mediated by reactive oxygen species (ROS), maintaining organelle function, and regulating intracellular signaling cascades.^42^, ^43^, ^44^ In the context of cancer, autophagy can play either a neutral, tumor‐suppressive, or tumor‐promoting role, depending on factors such as nutrient availability, microenvironmental stress, and pathological conditions.^45^ Apoptosis is a programmed form of self‐death that plays an important role in tumorigenesis. It is mediated by multiple signaling pathways and triggered by multiple factors, including cellular stress response, DNA damage, and immune detection. Some studies suggest that autophagy inhibits tumor growth by regulating ROS to prevent tumor initiation under oxidative stress conditions.^46^

Essentially, apoptosis and autophagy have many similarities in the process; they will activate the same signaling pathway and have similar protein composition. They can be triggered in the same cell at the same time as the tumor develops. In some cases, apoptosis inhibits autophagy or autophagy causes apoptosis. For example, emodin is an active ingredient in many Chinese herbal medicines; it can cause CRC cell apoptosis by inducing autophagy. There are some ATG genes that not only play a role in autophagy induction but also participate in apoptosis. For example, silencing of the Atg5 gene resulted in inhibition of autophagy and apoptosis, thus indicating an enhanced role of autophagy in inducing apoptosis.^47^, ^48^ In addition, some studies have suggested that autophagy promotes apoptosis by depleting endogenous inhibitors of cell death. As a tumor oncogene, SQSTM1/p62 is often abnormally upregulated and involved in the development of CRC. Some studies have pointed out that the expression of SQSTM1/p62 in CRC cells is inhibited by the β‐catenin/transcription factor (TCF) 4 complex, blocking phagocytosis.^49^ Therefore, in this study, under high‐glucose conditions, the cells are in a nutrient‐rich environment, and various genes and pathways involved in metabolism are in a continuously activated state, which puts the cells in a state of high‐speed operation, it will inhibit the execution of programmed cell death, such as apoptosis. Therefore, when the cells were in the state of autophagy inhibition, the apoptosis of the cells was also inhibited. The same conclusion was observed after the use of autophagy inhibitors (CQ), so autophagy regulated the apoptosis of tumor cells.

On the other hand, other research indicates that autophagy promotes cancer development. Mutations in RAS increase autophagy, enhancing tumor growth, survival, and aggressiveness.^50^ Mice with knockout of ATG5 and ATG7, essential autophagy genes, can develop liver cancer when exposed to oxidative stress and mitochondrial damage‐induced loss of autophagy.^51^, ^52^ Autophagy involves the delivery of cellular material to lysosomes for degradation, providing essential energy sources to cancer cells and promoting critical processes in tumor development, such as EMT. Autophagy can extensively coordinate and regulate the EMT process through different pathways.

Autophagy is mainly regulated by PI3K/AKT/mTOR, Beclin1, p53, and JAK/STAT signals. These regulatory pathways also have significant effects on EMT. The PI3K/AKT/mTOR pathway can activate mTOR to inhibit autophagy.^53^ This is consistent with the conclusion of this study. The PI3K/AKT/mTOR pathway also affects the EMT process. mTOR is an important molecule involved in autophagy process. When mTOR is activated, phosphorylation of ribosomal protein S6 (P70S6) will promote mRNA translation and inhibit endoplasmic reticulum delivery and autophagic membrane formation. It is worth noting that autophagy was activated when the mTOR pathway was inhibited, thereby inhibiting the EMT process of gallbladder cancer.^54^ Water stress protein (WSP1) induces autophagy by downregulating the PI3K/AKT/mTOR pathway and can reduce β‐catenin, and inhibits EMT by increasing E‐cadherin and reducing N‐cadherin, thereby inhibiting cancer migration.^55^ Studies have shown that metformin can inhibit mTOR signaling in thyroid cancer cells, while activating autophagy, resulting in the inhibition of cell proliferation and migration.^54^ This is consistent with the situation in this study. In high‐glucose conditions, mTOR signaling is activated, causing CRC cells to proliferate and migrate, so metformin can be used to restore this situation.

The relationship between high glucose and autophagy has also been preliminarily studied in other types of tumors. Zhou Cancan's study pointed out that the SREBP1‐autophagy axis can have a huge impact on the physiological function of pancreatic cancer cells when induced by a high‐glucose microenvironment.^56^ Chenyuan Li found that high glucose promoted breast cancer progression both in vitro and in vivo by upregulating MEDAG expression. Furthermore, MEDAG deficiency led to an increase in the number of autophagosomes and autophagic flux.^25^ Therefore, autophagy and EMT have such a regulatory relationship in various cancers; the prospect of utilizing autophagy to regulate the EMT process and consequently control tumor metastasis represents a promising discovery.

Since high glucose can inhibit autophagy and promote the proliferation and EMT of CRC cells, the tumor microenvironment of the proliferated CRC cells will also change. When the cells are in an environment with high glucose, it usually requires a stronger tolerance of the immune response, which was initially explored in this study.

Under the influence of high glucose, the expression of the inflammatory cytokines IL‐4 and IL‐13 increased, which is consistent with the results of Qiuping Zhao's research.^57^ Meanwhile, the expression of IL‐5 and TNF‐α decreased. IL‐4 and IL‐13 are structurally and functionally related Th2 cell cytokines.^58^ In normal physiological regulation and tumors, they both play roles in modulating immune responses. When IL‐4 and IL‐13 form ligand–receptor complexes, they activate signal transduction, which affects biological processes such as tumor cell proliferation, adhesion, and metastasis.^59^ IL‐4 plays multiple important roles in the tumor microenvironment. Research indicates that in mouse models of colon cancer and breast cancer, blocking IL‐4 can reduce the generation of immunosuppressive M2 phenotypes and MDSCs in the specific tumor microenvironment, and improve tumor‐specific CD8^+^ T cell responses.^60^ Additionally, blocking IL‐4 can enhance the responsiveness to anti‐OX40 immunotherapy.^61^ TNF‐α has been found to have tumoricidal effects both in vitro and in vivo. When TNF‐α binds to TNFR, it leads to an increase in lysosome levels within tumor cells. After the lysosomal uptake by target cells, lysosomal stability decreases, resulting in cellular autolysis, causing tumor hemorrhagic necrosis.^62^ TNF‐α plays a regulatory role in the body's immune system by promoting the expression of T cells and other cytotoxic cells, which can lead to the destruction or inhibition of tumor cells. In this study, there were no significant changes observed in IL‐12 levels. Research has shown that in mouse experiments, IL‐12 causes CD4^+^ T cells to differentiate to Th1 rather than Th2.^63^, ^64^ Therefore, it is believed that there is a dynamic interplay between IL‐4, IL‐13, and TNF‐α, which may explain the lack of significant changes in IL‐12 levels.

## 5 CONCLUSION

This study demonstrates that high glucose inhibits autophagy in CRC cells through the PI3K/AKT/mTOR pathway, leading to enhanced proliferation and suppression of apoptosis. These findings provide novel insights into how high glucose contributes to the initiation and progression of cancer.

## AUTHOR CONTRIBUTIONS

**Feng Li:** Investigation (equal); methodology (equal); writing – original draft (equal); writing – review and editing (equal). **Xing Wan:** Methodology (equal); supervision (equal). **Zhigui Li:** Investigation (equal); supervision (equal). **Liming Zhou:** Formal analysis (equal); project administration (equal); supervision (equal); writing – review and editing (equal).

## FUNDING INFORMATION

No funding was obtained for this study.

## CONFLICT OF INTEREST STATEMENT

The authors declare that they have no competing interests.

## ETHICS APPROVAL AND CONSENT TO PARTICIPATE

The study was approved by the Animal Ethics Committee of Sichuan University.

## CONSENT FOR PUBLICATION

Not applicable.

## Supporting information


Appendix S1.

## Data Availability

All data generated or analyzed during this study are included in this article.
