# Supplementary material for: High glucose inhibits autophagy and promotes the proliferation and metastasis of colorectal cancer through the PI3K/AKT/mTOR pathway
Source: Cancer Med. 2024 Jun 13;13(11):e7382. doi: 10.1002/cam4.7382 (PMC11176572; doi:10.1002/cam4.7382)
Supplement: Supplementary file 1 — Appendix S1. [file CAM4-13-e7382-s001.docx]

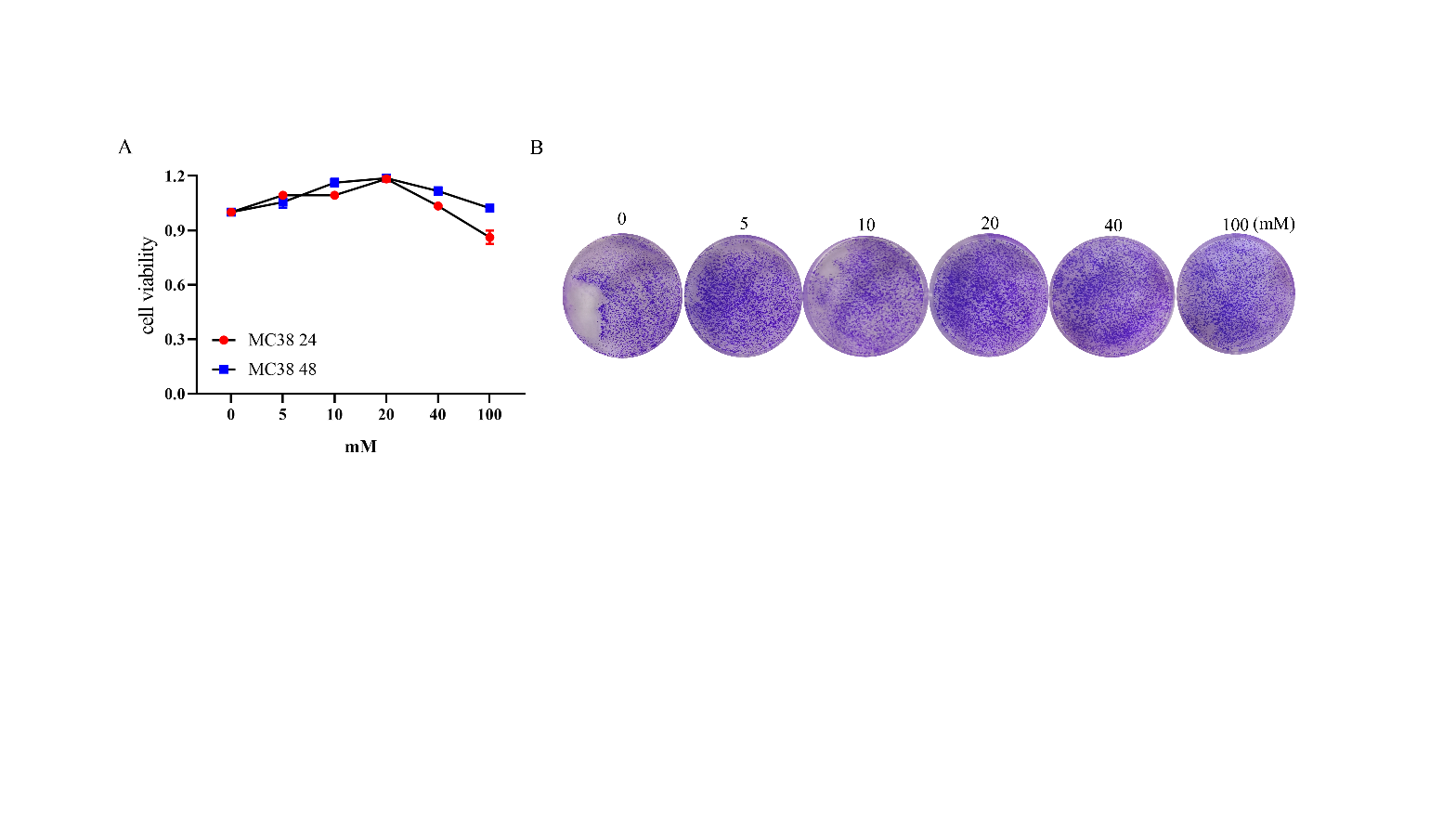


Figure S1 High glucose promotes MC38 cells proliferation.


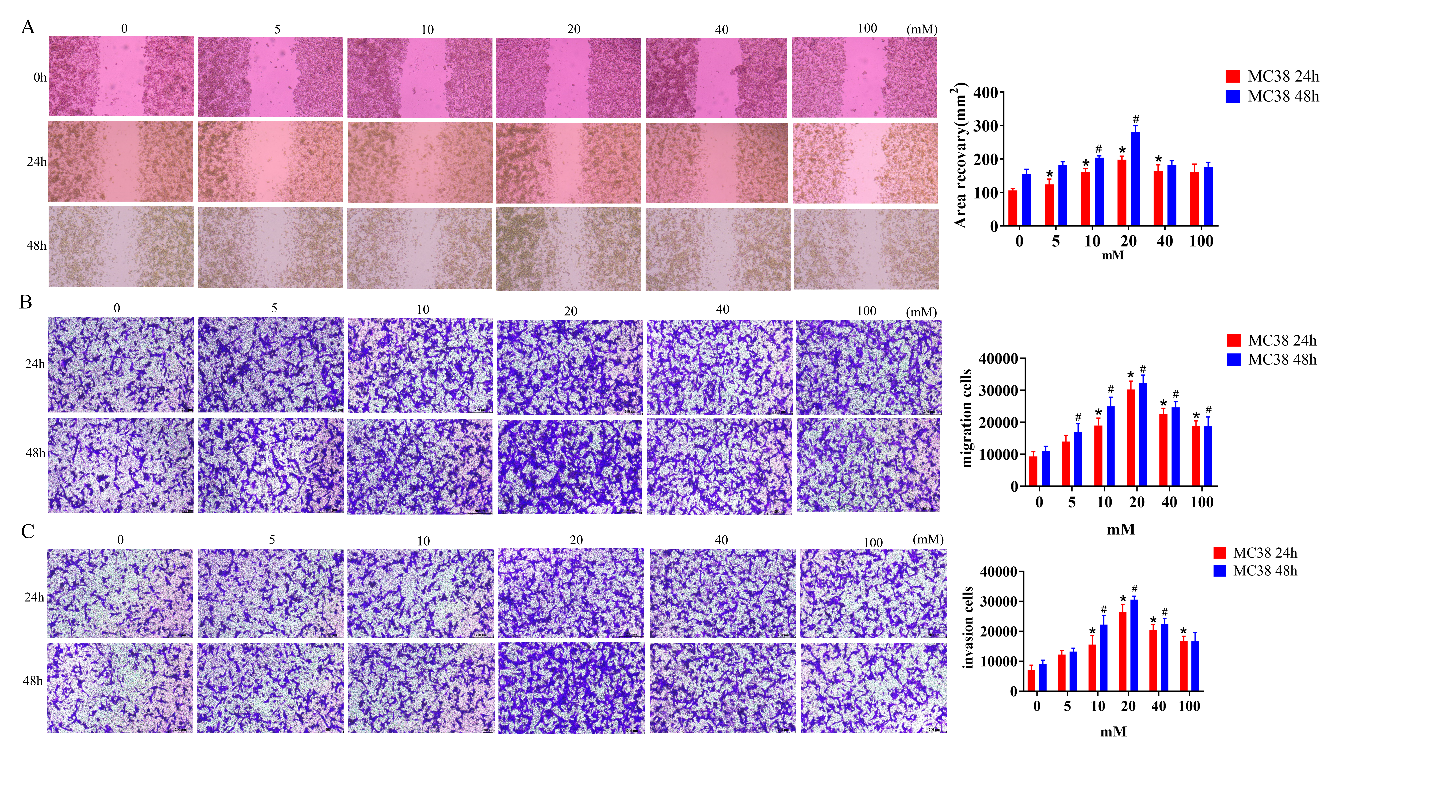


Figure S2 High glucose promotes MC38 cells migration.


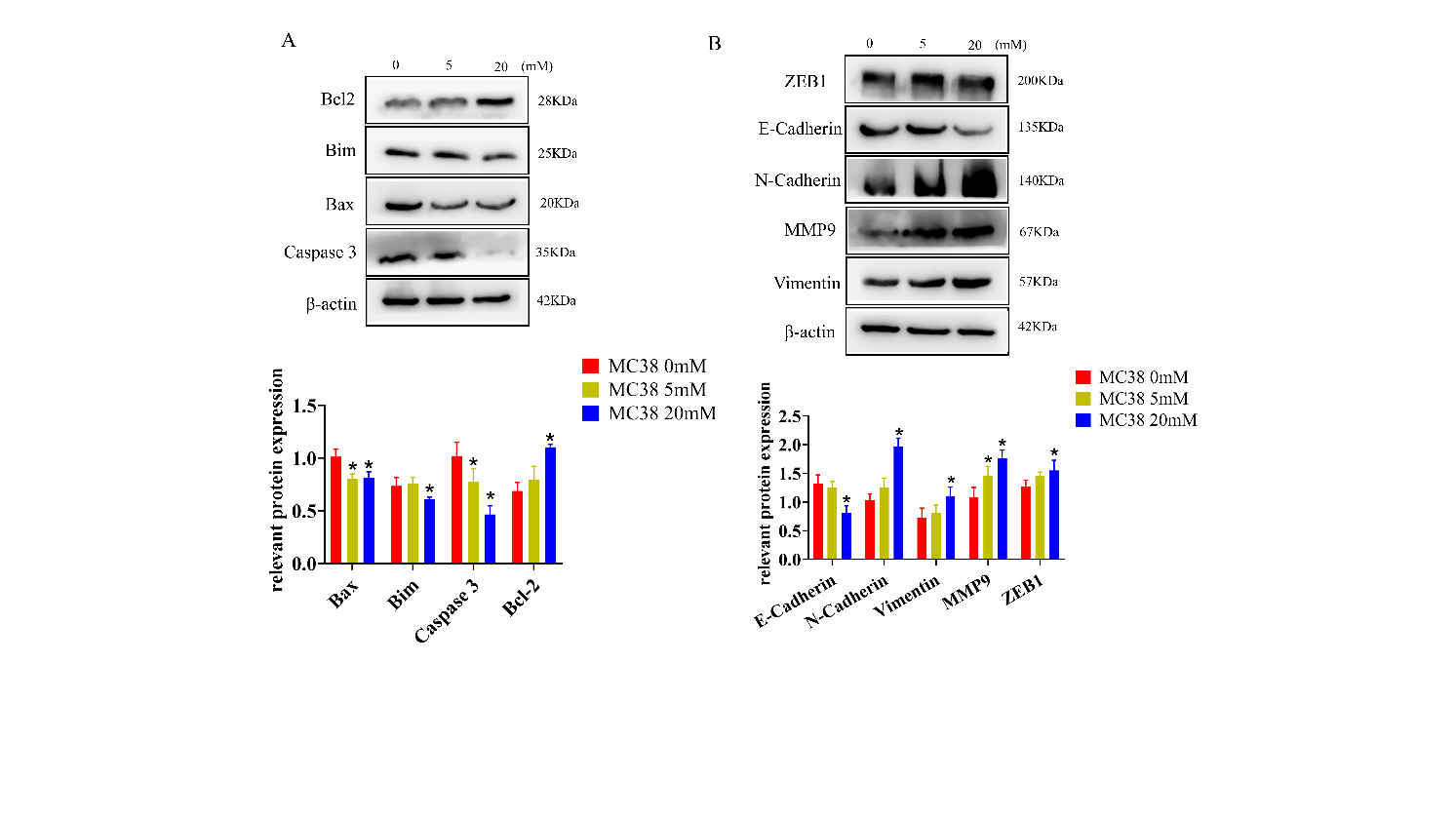


Figure S3 High glucose suppresses apoptosis-related protein expression and promotes EMT-related protein expression in MC38 cells.


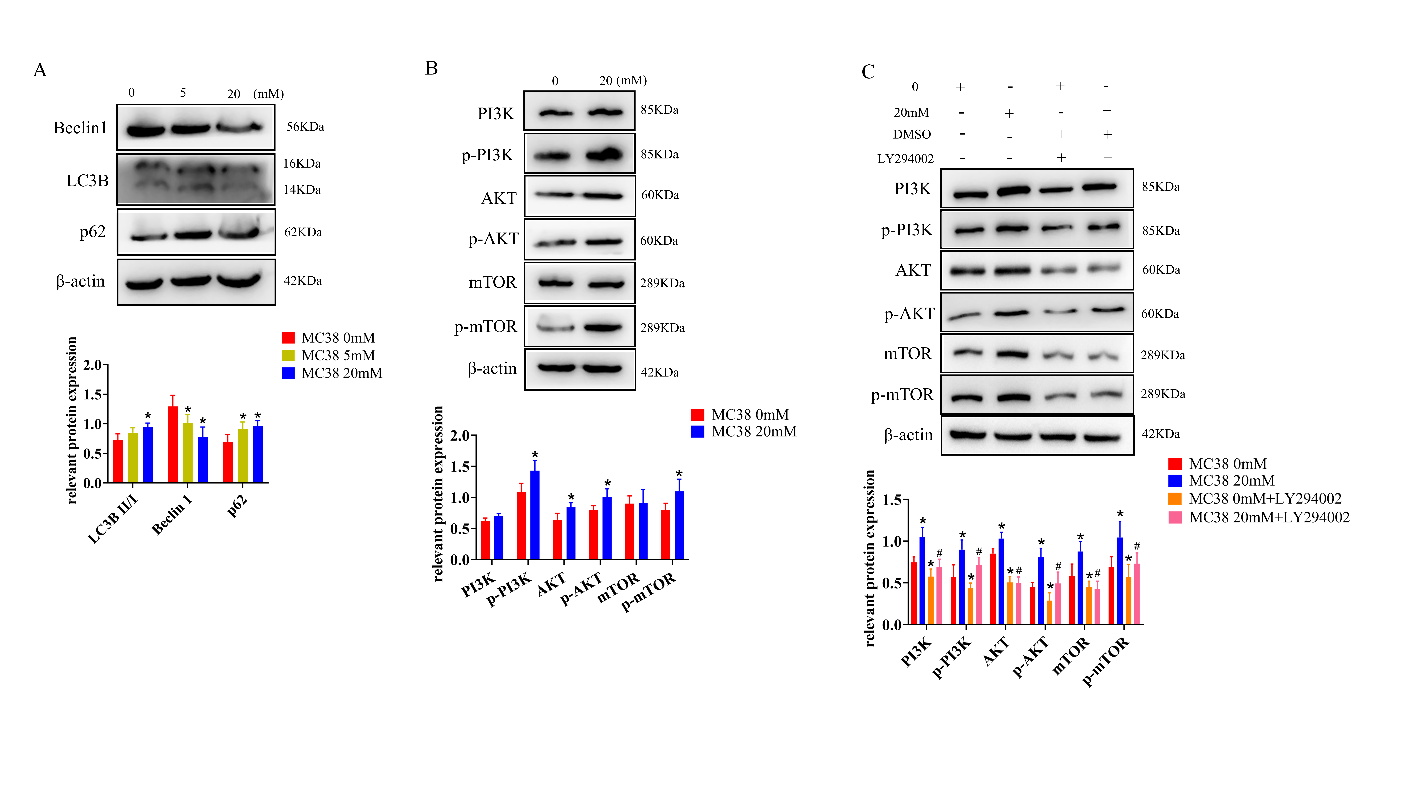


Figure S4 High glucose suppresses autophagy-related gene and enhances the expression of PI3K/AKT/mTOR pathway proteins in MC38 cells

Table S1 Antibodies information

| Antibody | Brand | Dilution ratio |
| --- | --- | --- |
| Bcl-2 | SAB | 1:1000 |
| Bax | CST | 1:1000 |
| Bim | CST | 1:1000 |
| Caspase-3 | CST | 1:1000 |
| n-cadherin | CST | 1:1000 |
| e-cadherin | CST | 1:1000 |
| MMP9 | proteintech | 1:1000 |
| ZEB1 | abcam | 1:1000 |
| vimentin | abcam | 1:1000 |
| P62 | MBL | 1:1000 |
| LC3 | CST | 1:1000 |
| Beclin1 | proteintech | 1:1000 |
| PI3K | abcam | 1:1000 |
| p-PI3K | abcam | 1:1000 |
| AKT | abcam | 1:1000 |
| p-AKT | abcam | 1:1000 |
| mTOR | proteintech | 1:1000 |
| p-mTOR | HUABIO | 1:1000 |
| β-actin | HUABIO | 1:2000 |
| KI67(for IHC) | abcam | 1:1000 |
